# Off-Label Underdosing or Overdosing of Non-vitamin K Antagonist Oral Anticoagulants in Patients With Atrial Fibrillation: A Meta-Analysis

**DOI:** 10.3389/fcvm.2021.724301

**Published:** 2021-09-08

**Authors:** Xiaojuan Wu, Linyan Hu, Jinjin Liu, Qiuping Gu

**Affiliations:** ^1^Department of Gastroenterology, Ganzhou People's Hospital, Ganzhou, China; ^2^Hengshui Health School, Hengshui, China; ^3^Department of Oncology, Ganzhou People's Hospital, Ganzhou, China

**Keywords:** atrial fibrillation, anticoagulants, off label, outcomes, meta-analysis

## Abstract

**Background:** Several studies have investigated the role of off-label non-vitamin K antagonist oral anticoagulants (NOACs) in patients with atrial fibrillation (AF). We aimed to compare the effectiveness and safety outcomes between off-label underdose or overdose vs. on-label dose of NOACs in AF patients.

**Methods:** The PubMed database was systematically searched until August 2021. Observational cohorts were included if they compared the outcomes of off-label underdose or overdose with on-label dose of NOACs in AF patients. The risk ratios (RRs) and 95% confidence intervals (CIs) were pooled using a fixed-effects model (*I*^2^ ≤ 50%) or a random-effects model (*I*^2^ > 50%).

**Results:** A total of 15 observational studies were included. Compared with on-label dose of NOACs, off-label underdose of NOACs was associated with increased risks of stroke or systemic embolism (RR = 1.09, 95% CI 1.02–1.16), and all-cause death (RR = 1.29, 95% CI 1.10–1.52) but not ischemic stroke (RR = 1.34, 95% CI 0.76–2.36), myocardial infarction (RR = 1.08, 95% CI 0.92–1.28), major bleeding (RR = 0.97, 95% CI 0.89–1.05), intracranial hemorrhage (RR = 1.12, 95% CI 0.90–1.40), and gastrointestinal bleeding (RR = 0.96, 95% CI 0.85–1.07), whereas off-label overdose of NOACs was associated with increased risks of SSE (RR = 1.20, 95% CI 1.05–1.36), all-cause death (RR = 1.22, 95% CI 1.06–1.39), and major bleeding (RR = 1.33, 95% CI 1.16–1.52) but not gastrointestinal bleeding (RR = 1.18, 95% CI 0.99–1.42) and myocardial infarction (RR = 0.98, 95% CI 0.75–1.30).

**Conclusion:** Compared with on-label dose of NOACs, off-label underdose was associated with increased risks of stroke or systemic embolism and all-cause death, whereas off-label overdose of NOACs was associated with increased risks of stroke or systemic embolism, all-cause death, and major bleeding.

## Introduction

Atrial fibrillation (AF) is the most common arrhythmia in clinical practice, affecting millions of people worldwide (1). Appropriate thromboprophylaxis with anticoagulants such as warfarin and non-vitamin K antagonist oral anticoagulants (NOACs) is an urgent need in general AF patients or patients with other specific conditions (2–4). Since the use of NOACs has improved the benefit-harm profiles for stroke prevention when compared with warfarin, several AF guidelines have regarded NOACs (dabigatran, rivaroxaban, apixaban, or edoxaban) as the first choice drug for stroke prevention in patients with AF (1, 5). A previous study (6) has summarized that the reduced dose of NOACs could be used in several circumstances. In real-world clinical practice, some users of reduced-dose NOACs do not conform to the label- or guideline- recommendations (i.e., off-label underdose of NOACs) (7). More recently, several studies have explored the effect of off-label underdose or overdose of NOACs in AF patients, but the corresponding findings are not completely consistent. Therefore, this meta-analysis was performed to assess the effectiveness and safety outcomes between off-label underdose or overdose vs. on-label dose of NOACs in patients with AF.

## Methods

### Literature Retrieval

This meta-analysis was performed according to the Preferred Reporting Items for Reporting Systematic Reviews and Meta-analyses (Supplementary Table 1). This was a meta-analysis of the published studies, and no ethical approval was warranted. The PubMed electronic database was systematically searched until August 2021 for the relevant studies. Supplementary Table 2 shows the detailed search strategies. The reference lists of the included studies were screened to identify the additional publications.

### Inclusion and Exclusion Criteria

Observational cohort studies were included if adult patients with non-valvular AF received at least off-label underdose or overdose of one NOAC (dabigatran, rivaroxaban, apixaban, or edoxaban). Effectiveness outcomes included stroke or systemic embolism (SSE), ischemic stroke (IS), myocardial infarction (MI), and all-cause death; and safety outcomes included major bleeding, intracranial hemorrhage (ICH), and gastrointestinal (GI) bleeding. The primary effectiveness outcome was SSE, and the primary safety outcome was major bleeding. We defined the reduced-dose NOACs that did not conform to the label- or guideline- recommendations as off-label underdose or overdose of NOACs. The on-label dose of NOACs was regarded as control. We excluded the study type such as reviews, case reports, case series, and meeting abstracts because they had no effect estimates.

### Data Extraction and Quality Assessment

Two authors independently finished the study selection and collected the following data: the first author and publication year, location, design of the study, inclusion period, data source, age and sex, type or dose of NOACs, and definitions of off-label underdose or overdose of NOACs in each included study.

The Newcastle-Ottawa Scale was applied to evaluate the quality of observational studies. This scoring tool had three domains including the selection of cohorts, the comparability of cohorts, and the assessment of the outcome. A study with a Newcastle-Ottawa Scale score of <6 points was defined as low quality.

### Data Analysis

The Cochrane Q test and *I*^2^ statistic were the most commonly reported statistical methods to assess the heterogeneity across the included studies. The results of included studies were expressed as the adjusted risk ratios (RRs) and 95% confidence intervals (CIs). Propensity score-matched or adjusted RRs and 95% CIs were abstracted from each included study. We calculated the natural logarithms of RRs and their standard errors of the included studies, which were pooled by a fixed-effects model (*I*^2^ ≤ 50%) or a random-effects model (*I*^2^ > 50%) using an inverse variance method. For the primary effectiveness and safety outcomes, the method of exclusion of one study at a time was applied in the sensitivity analysis. The publication bias was assessed using the funnel plots.

All of the statistical analyses were performed using the Review Manager 5.3 software (the Nordic Cochrane Center, Rigshospitalet, Denmark).

## Results

### Study Selection and Patients' Characteristics

**Figure 1:**
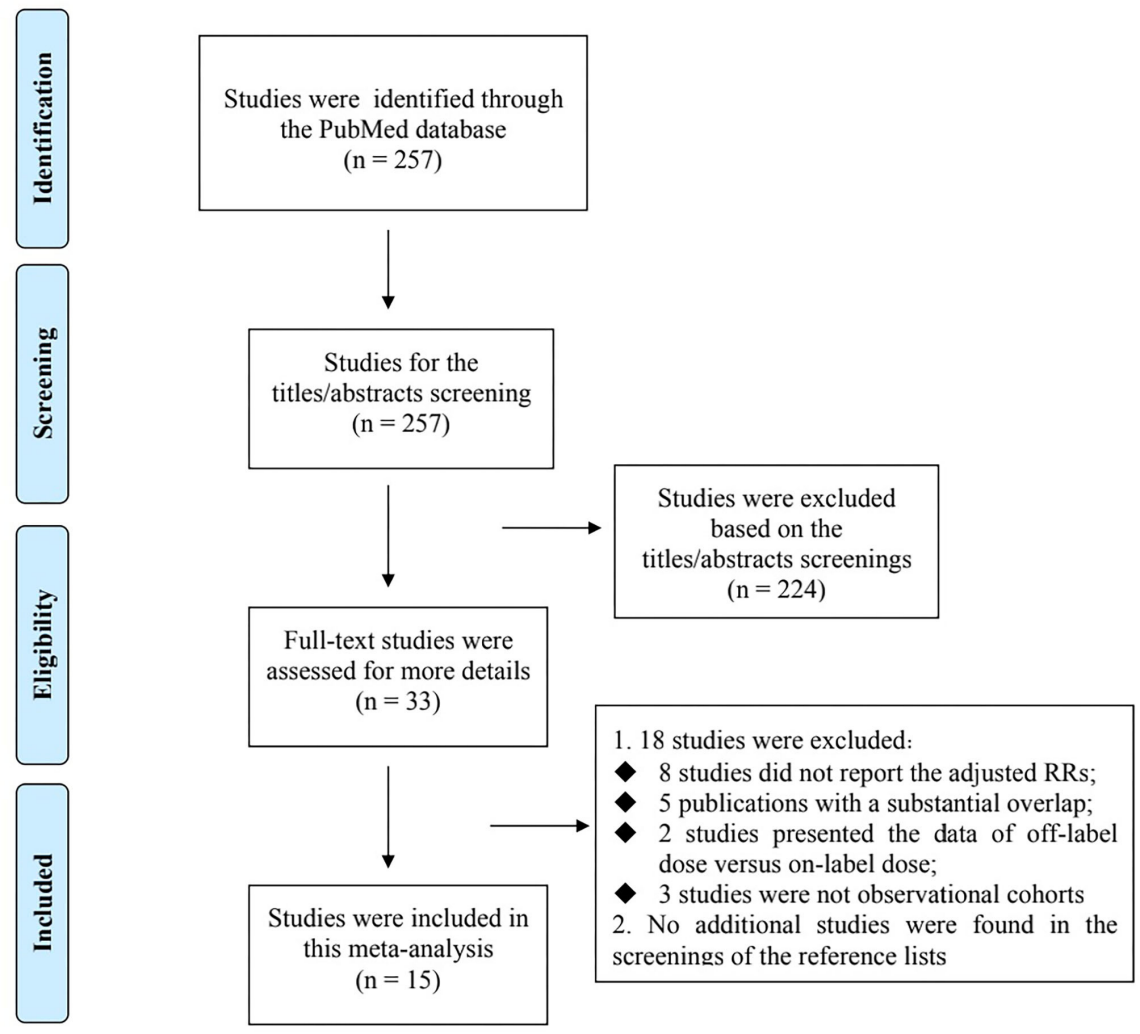
Flowchart of electronic retrievals of this meta-analysis.

Flowchart of electronic retrievals is presented in Figure 1. A total of 257 records from the PubMed database were identified, and 33 full-text studies were reviewed for more details. Among them, 18 studies were excluded because: (1) eight studies (8–15) did not report the adjusted RRs; (2) five individual studies have overlapping data (16–20) (3) two studies presented the data of off-label dose (including overdosing and underdosing) vs. on-label dose of NOACs (21, 22), and (4) three studies were not observational cohorts (23–25). No additional studies were found in the screenings of the reference lists of the relevant studies. Finally, 15 observational cohort studies were included in this meta-analysis (26–40). The baseline characteristics of the 15 included studies are presented in Table 1. All of the included studies had an acceptable quality with the Newcastle-Ottawa Scale score of >6 points.

**Table 1:** Baseline characteristics of the included studies.

**References**	**Location**	**Study period; design**	**Population source**	**Number of patients**	**Mean age (y)/Sex**	**NOACs (type and dosing)**	**NOS tool**
Cheng et al. (34)	Taiwan	2012–2016; cohort	Taipei Veterans General Hospital	2,214	75.7/both	Underdosing: Rivaroxaban-10 mg	7
Chen et al. (27)	Taiwan	2014–2018; cohort	4 hospitals in southern Taiwan	1,073	75.1/both	Underdosing: apixaban-2.5 mg	7
Chan et al. (31)	Taiwan	2011–2018; cohort	Chang Gung Memorial Hospital	11,275	74.2/both	Underdosing: dabigatran-110 mg; rivaroxaban-15 or 10 mg; apixaban-2.5 mg; edoxaban-30 mgOverdosing: dabigatran-150 mg; rivaroxaban-20 mg; apixaban-5 mg; edoxaban-60 mg	8
Arbel et al. (37)	Israel	2011–2017; cohort	Clalit Health Services	8,245	76.5/both	Dabigatran, rivaroxaban, apixaban; unknown doses	7
Ikeda et al. (35)	Japan	NR; cohort	XAPASS	6,159	69.2/both	Underdosing: Rivaroxaban-10 mg	7
Murata et al. (36)	Japan	2013–2015;cohort	SAKURA AF	1,115	69.0/both	Underdosing: Dabigatran-110 mg, rivaroxaban-10 mg, apixaban-2.5 mg, edoxaban-30 mg	7
Ohno et al. (29)	Japan	2011–2017; cohort	DIRECT registry	2,216	71.6/both	Underdosing: dabigatran-110 mg; rivaroxaban-15 or 10 mg; apixaban-2.5 mg; edoxaban-30 mgOverdosing: dabigatran−150 mg; rivaroxaban-20 mg; apixaban-5 mg; edoxaban-60 mg	8
Yao et al. (38)	United States	2010–2015; cohort	OptumLabs Data Warehouse	3,554	NR/both	Dabigatran, rivaroxaban, apixaban; unknown doses	8
Steinberg et al. (40)	United States	2013–2016; cohort	ORBIT-AF II	5,738	71.0/both	Underdosing: Dabigatran-75 mg, rivaroxaban-15 mg, apixaban-2.5 mgOverdosing: dabigatran−150 mg; rivaroxaban-20 mg; apixaban-5 mg	8
Briasoulis et al. (33)	United States	2010–2016; cohort	Medicare beneficiaries	8,035	NR/both	Underdosing: Dabigatran-75 mg; rivaroxaban-15 mg	7
Ashraf et al. (28)	United States	2001–2017; cohort	3 Mayo Clinic sites	8,125	73.3/both	Underdosing: Dabigatran-75 mg, rivaroxaban-15 mg, apixaban-2.5 mg, edoxaban-30 mg	8
Lee et al. (39)	Korea	2012–2013; cohort	Chonnam National University Hospital	366	NR/Both	Underdosing: Dabigatran-110 mg	7
Yu et al. (30)	Korea	2013–2016; cohort	Korean National Health Insurance Service database	53,649	70.5/Both	Dabigatran, rivaroxaban, apixaban, edoxaban; unknown doses	8
Camm et al. (32)	Multicenter, 35 countries	2013–2016; cohort	Global Anticoagulant Registry in the FIELD-AF	10,426	74.0/both	Underdosing: Dabigatran-110 mg (EMA) or 75 mg (FDA); rivaroxaban-15 mg; apixaban-2.5 mg; edoxaban-30 mg	8
Fernandez et al. (26)	Spain	NA; cohort	EMIR	1,421	74.2/both	Underdosing: rivaroxaban-15 mg	7

### Effectiveness and Safety of Off-Label Underdose vs. On-Label Dose of NOACs

**Figure 2:**
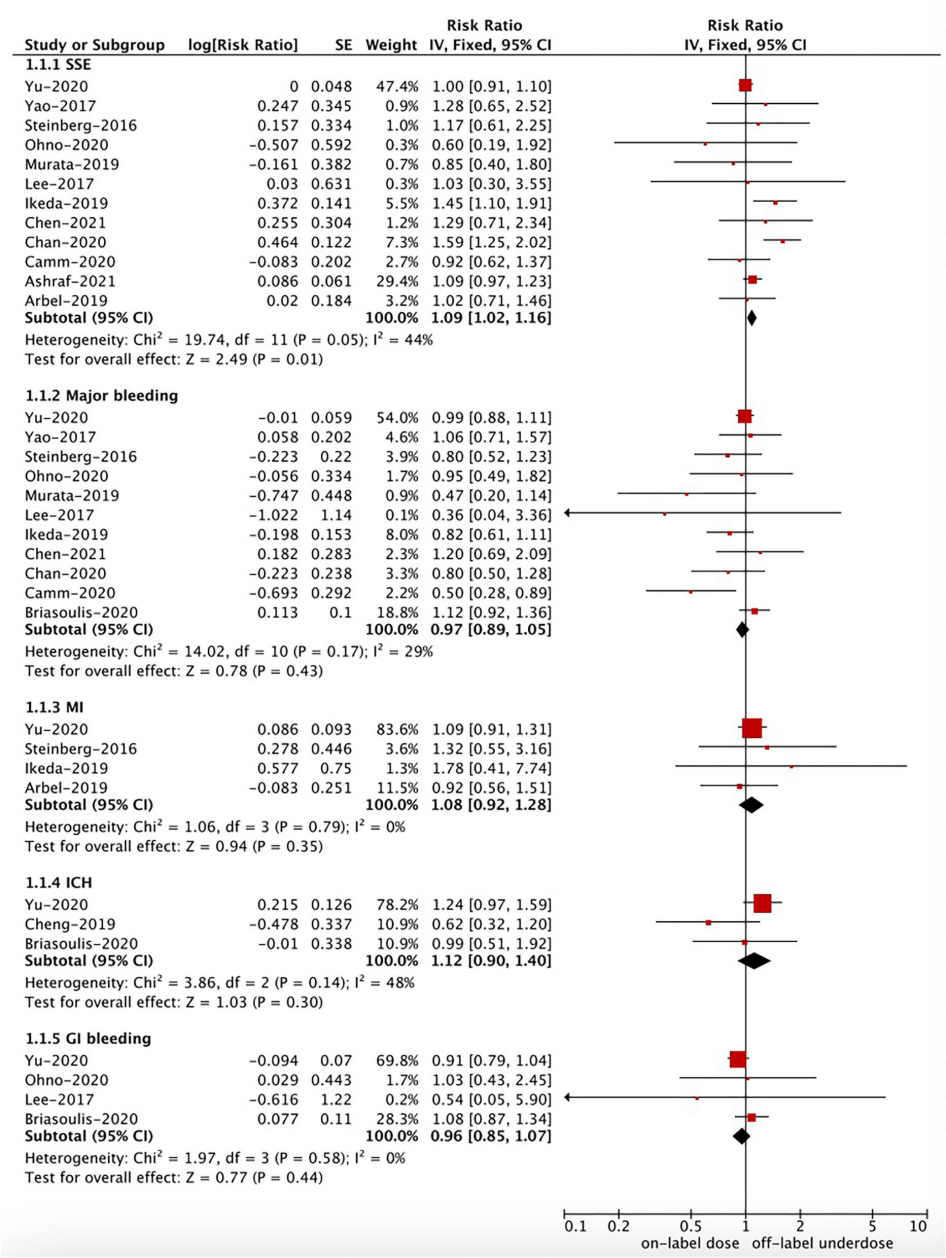
A fixed-effects model for comparing the outcomes between off-label underdose and on-label dose of NOACs in patients with atrial fibrillation. NOACs, non-vitamin K antagonist oral anticoagulants; SSE, stroke or systemic embolism; IS, ischemic stroke; MI, myocardial infarction; ICH, intracranial hemorrhage; GI, gastrointestinal; CI, confidence interval; SE, standard error; IV, inverse of the variance.

**Figure 3:**
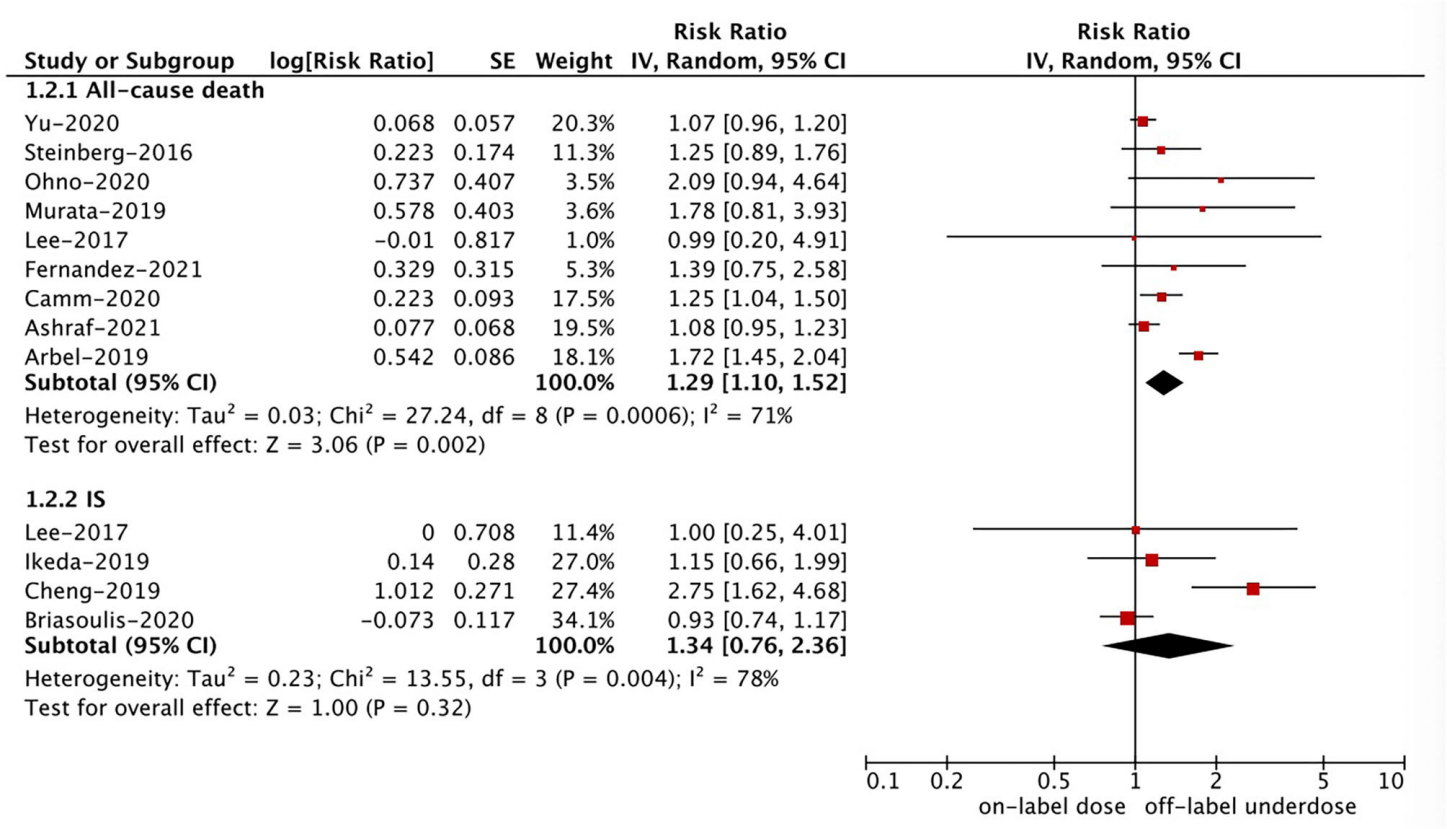
A random-effects model for comparing the outcomes between off-label underdose and on-label dose of NOACs in patients with atrial fibrillation. NOACs, non-vitamin K antagonist oral anticoagulants; IS, ischemic stroke; CI, confidence interval; SE, standard error; IV, inverse of the variance.

As shown in Figures 2, 3, in the pooled analysis, compared with on-label dose of NOACs, off-label underdose of NOACs was associated with increased risks of SSE (RR = 1.09, 95% CI 1.02–1.16; *P* = 0.01; *I*^2^ = 44%), and all-cause death (RR = 1.29, 95% CI 1.10–1.52; *P* = 0.002; *I*^2^ = 71%). There were no significant differences in the rates of IS (RR = 1.34, 95% CI 0.76–2.36; *P* = 0.32; *I*^2^ = 78%), MI (RR = 1.08, 95% CI 0.92–1.28; *P* = 0.35; *I*^2^ = 0%), major bleeding (RR = 0.97, 95% CI 0.89–1.05; *P* = 0.43; *I*^2^ = 29%), ICH (RR = 1.12, 95% CI 0.90–1.40; *P* = 0.30; *I*^2^ = 48%), and GI bleeding (RR = 0.96, 95% CI 0.85–1.07; *P* = 0.44; *I*^2^ = 0%) between off-label underdose vs. on-label dose of NOACs.

### Effectiveness and Safety of Off-Label Overdose vs. On-Label Dose of NOACs

**Figure 4:**
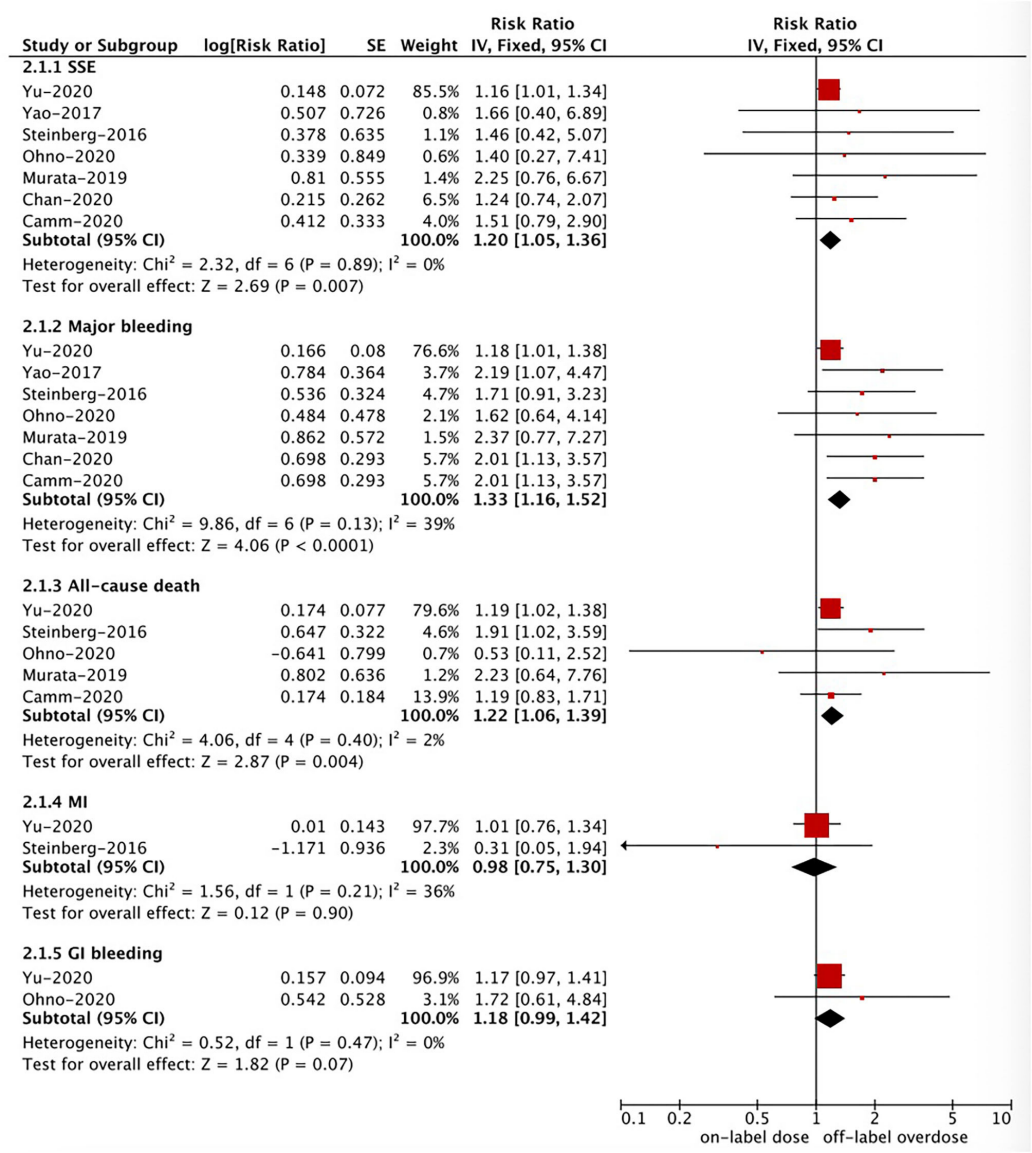
A fixed-effects model for comparing the ischemic stroke between off-label overdose and on-label dose of NOACs in patients with atrial fibrillation NOACs, non-vitamin K antagonist oral anticoagulants; SSE, stroke or systemic embolism; MI, myocardial infarction; CI, confidence interval; SE, standard error; IV, inverse of the variance.

Based on the pooled results shown in Figures 3, 4, compared with the on-label dose of NOACs, off-label overdose of NOACs was associated with increased risks of SSE (RR = 1.20, 95% CI 1.05–1.36; *P* = 0.007; *I*^2^ = 0%), all-cause death (RR = 1.22, 95% CI 1.06–1.39; *P* = 0.004; *I*^2^ = 2%), and major bleeding (RR = 1.33, 95% CI 1.16–1.52; *P* < 0.0001; *I*^2^ = 39%), but had similar risks of GI bleeding (RR = 1.18, 95% CI 0.99–1.42; *P* = 0.07; *I*^2^ = 0%) and MI (RR = 0.98, 95% CI 0.75–1.30; *P* = 0.90; *I*^2^ = 36%). No pooled data for IS and ICH in this section due to the limited studies.

### Sensitivity Analysis and Publication Bias

For the primary effectiveness and safety outcomes, after we excluded one study at a time, the corresponding results did not change substantially. As shown in Supplementary Figures 1, 2, there were seemingly no obvious publication biases assessed using the funnel plots.

## Discussion

In the present study, our results suggested that compared with the on-label dose of NOACs, the use of off-label underdose of NOACs was associated with increased risks of SSE and all-cause death but not IS, MI, and bleeding outcomes, whereas the use of off-label overdose of NOACs was associated with increased risks of SSE, all-cause death, and major bleeding but not MI and GI bleeding. Our current evidence further support the findings of previous meta-analyses (41, 42) by adding the latest studies, suggesting the importance of appropriate NOAC dosing according to the NOAC-dose adjustment criteria.

Despite the greater net clinical benefit of NOACs compared with warfarin among AF patients, there are still many patients who do not receive appropriate oral anticoagulant therapy. Recent observational data have shown that the NOAC dosing that is inconsistent with the label- or guideline-recommendations are becoming a widespread phenomenon (38, 43, 44). Inappropriate NOAC dosing in routine clinical practice is a serious concern and may be associated with an increase in the risk of adverse events. The reasons why physicians select the underdose of NOACs remain unclear. Patients in real-world settings are often sicker and more fragile than those in the NOAC trials. These sicker and more fragile patients might have a higher risk of bleeding, which is a potential reason for the NOAC underdosing. A current study has demonstrated that a substantial proportion of the NOAC underdosing may be voluntary, indicating a cautious approach to patients perceived to be at high risk of bleeding (27). The authors proposed that patients' characteristics (e.g., advanced age, previous bleedings, comorbidities), rather than the intensity of oral anticoagulant therapy, are associated with an increase in the bleeding risks (27).

In real-world settings, physicians are afraid of bleeding events induced by the anticoagulation treatment in patients with AF. As such, they tend to prescribe a reduced dose of NOACs with no indications. However, whether the NOAC underdosing could be the correct dosing for most of the NOAC users is unclear. Reduced dose regimens of NOACs might reduce the bleeding risk at a cost of the increased risk of AF-related thromboembolic events due to under exposure. A prior systematic review has concluded that AF patients treated with an off-label dose of NOACs did not acquire the full benefits of the anticoagulation treatment, and might increase the risks of stroke and bleeding events (6). However, this was only a descriptive analysis because limited quantitative data were linking NOAC-dose adjustments and adverse outcomes at that time. Given pooling of different data sources could improve the generalizability of research findings, we conducted a meta-analysis by including more studies to determine the effect of off-label underdose of NOACs in patients with AF. Our current evidence indicated that compared with the on-label dose of NOACs, the use of off-label underdose was associated with increased risks of SSE and all-cause death. Of note, a case-control study by Paciaroni et al. (23) focused on a specific population (i.e., AF patients who had an acute cerebrovascular ischemic event); and the outcome was a recurrent stroke. Therefore, we performed a descriptive analysis for the study of Paciaroni et al. (23). In this study, off-label underdose of NOACs was associated with an increased risk of recurrent stroke (RR = 3.18, 95% CI 1.95–5.85). As such, it is important to have an appropriate NOAC dosing according to the label- or guideline- recommendations. Previous studies indicated that bleeding could be a problem in AF patients treated with the underdose of NOACs (6, 45, 46). However, these studies did not account for differences in patient characteristics with adjustments. Our current meta-analysis only included the studies with adjusted data, suggesting that the incidence rate of major bleeding was similar between off-label underdose vs. on-label dose of NOACs. Inconsistent with the findings from the study of Bo et al., the use of off-label underdose of oral factor Xa inhibitors do not provide a sizeable net clinical benefit, but rather has increased risks of adverse events including hospitalizations for cardiovascular causes and stroke, without a reduced risk of bleeding (27).

In the study of Yao et al. (38) inappropriate reduced doses of apixaban reduced the effectiveness of stroke prevention, manifesting as a nearly 5-fold increased rate of stroke. Interestingly, the decreased effectiveness associated with an inappropriate reduced dose of apixaban was not found in AF patients treated with off-label underdose of dabigatran or rivaroxaban. Off-label underdose of rivaroxaban (34) increased the risk of IS, but dabigatran did not (39). Of note, the number of included studies in the subgroup analysis based on the type of NOACs was relatively small, which would limit the validity of the corresponding findings. Future prospective, dedicated, observational real-life studies should further shed some light on the potential clinical benefit of the off-label underdose of NOACs (considering the type of NOACs) in selected patients. In addition, further study would be improved if we could be more sure as to whether the underdosing of NOACs is due to overestimated bleeding risks, underestimated stroke risks, and patient's frailty. It is important to know why a practitioner might reasonably reduce the NOAC dosing. It is also important that not only the percentage dose reduction relative to theoretically ideal daily dosage be nominated, but also an increase in the inter-dose interval. Further clinical researches could pay close attention to these issues.

### Limitations

Several limitations were noted in this meta-analysis. First, several unmeasured factors might exist in real-world studies, which could be the residual confounders. In addition, we only searched the PubMed database for relevant studies. Second, we did not perform the subgroup analysis based on the patient's age, sex and other information due to the limiting data. Third, information such as the adherence or persistence to NOACs were not considered.

## Conclusions

Current evidence indicated that compared with the on-label dose of NOACs, the use of off-label underdose was associated with increased risks of SSE and all-cause death, whereas off-label overdose of NOACs was associated with increased risks of SSE, all-cause death, and major bleeding.

## Data Availability Statement

The original contributions generated for the study are included in the article/Supplementary Material, further inquiries can be directed to the corresponding authors.

## Author Contributions

All authors listed have made a substantial, direct and intellectual contribution to the work, and approved it for publication.

## Conflict of Interest

The authors declare that the research was conducted in the absence of any commercial or financial relationships that could be construed as a potential conflict of interest.

## Publisher's Note

All claims expressed in this article are solely those of the authors and do not necessarily represent those of their affiliated organizations, or those of the publisher, the editors and the reviewers. Any product that may be evaluated in this article, or claim that may be made by its manufacturer, is not guaranteed or endorsed by the publisher.
